# Copy number variations in ***AMY2B*** gene and amylase activity in Balkan dog breeds

**DOI:** 10.1371/journal.pone.0322775

**Published:** 2025-05-02

**Authors:** Jasmin Katica, Teufik Goletić, Aida Kavazović, Maja Varatanović, Ćazim Crnkić

**Affiliations:** Department of Animal Production and Biotechnology, University of Sarajevo – Veterinary Faculty, Sarajevo, Bosnia and Herzegovina; Banaras Hindu University, INDIA

## Abstract

This study aims to estimate the number of *AMY2B* gene copies and measure serum amylase activity in several Balkan dog breeds. Additionally, it explores the relationship between these genetic and biochemical parameters. Blood samples from 85 dogs representing eight breeds were collected, DNA was extracted, and *AMY2B* copy numbers were determined using droplet digital PCR. *AMY2B* gene copies ranged from 7.7 to 18.4, with a mean of 12.4 ± 2.2. Significant breed-related differences were observed (p = 0.025), with Istrian Wire-Haired Hounds showing the highest mean copy number (13.9 ± 1.5) and Posavatz Hounds the lowest (10.8 ± 1.5). Serum amylase activity ranged from 3.3 to 17.8 µkat/L, with a mean of 8.7 ± 2.6, and showed significant interbreed differences (p = 0.004), with Barak breed displaying the highest activity. Serum glucose levels varied widely, but no significant interbreed differences were detected (p = 0.340). No significant correlation was found between *AMY2B* copy numbers and serum amylase activity or glucose levels. The study concludes that Balkan dogs have *AMY2B* copy numbers similar to other European breeds, likely reflecting historical agricultural practices in the region, thereby facilitating better starch digestion. While significant variations exist among breeds, the lack of correlation between gene copy number and amylase activity suggests that other factors influence enzyme levels.

## Introduction

Throughout millennia of dog domestication, there have been changes in their dietary habits, with a trend towards increasingly utilizing plant-based foods. Today, dogs commonly consume food containing relatively large amounts of starch, a typical plant carbohydrate, which they digest very efficiently [1] and without proven negative consequences on health and well-being [2]. This supports the hypothesis that dogs, on their evolutionary path from wolf ancestors, alongside morphological and behavioral changes, have also adapted their physiological and dietary characteristics to coexist with humans and utilize food leftovers rich in starch [3].

Recent studies have demonstrated that dog domestication involved genetic changes related to the utilization of food, especially plant carbohydrates, which were not a significant part of the wolf’s diet. Specifically, there has been an expansion observed in three genes with key roles in starch digestion, including α-amylase 2B (*AMY2B*), maltase-glucoamylase (MGAM), and sodium-glucose transporters (SGLT1) [3]. Indeed, one of the striking genetic distinctions observed between dogs and wolves is the significantly increased number of copies of the *AMY2B* gene in dogs responsible for α-amylase production in pancreas. Amylase is enzyme crucial for starch digestion, and the higher *AMY2B* copy number in dogs suggests an evolutionary adaptation for enhanced carbohydrate processing compared to their wolf ancestors. Wolves typically possess two copies of this gene, whereas studies have revealed that dogs exhibit a wider range, from four to 30 copies [3] and even more than that in some cases [4,5]. This variation implies varying levels of starch-digesting capabilities among dogs, potentially differing both between and within breeds [4,6]. A direct experimental comparison of starch-digesting capabilities among dogs with different numbers of *AMY2B* genes has not been conducted, but it is believed that breeds with a higher number of the genes are better adapted to starch utilization due to increased amylase activity.

It has been found that ancient dogs originating from hunting societies and regions with no, or recent, agricultural practices exhibit a lower number of *AMY2B* compared to those domesticated in regions with earlier development of prehistoric agriculture [4–7]. Generally speaking, farming has led to a multiple-fold increase in the number of copies of the gene encoding pancreatic amylase in most dog breeds [6]. However, dogs from areas where the climate was not suitable for large-scale agriculture, and where fish and meat likely formed a greater proportion of the diet, had a lower number of copies since they did not benefit from the gene duplication [4].

Dog breeds of European origin have been investigated in previous studies [3,5,6,8], but none of these studies included native Balkan breeds. Given that the broader Balkan region (Southeastern Europe) is part of an area of early agricultural development [8,9], it was hypothesized that dogs from this region have a relatively high number of copies of the *AMY2B* gene and are well-adapted to dietary starch utilization. The aim of this study was to estimate *AMY2B* copy numbers and serum amylase activity in several dog breeds originating from the Balkans and also to examine the relationship between these two parameters, as well as their association with the serum glucose levels in dogs.

## Materials and methods

The present study was conducted on blood samples from a total of 85 dogs across eight native Balkan breeds. The following breeds were included: Bosnian Broken-Haired Hound - Barak (BAR) n = 14, Posavatz Hound (POS) n = 13, Istrian Short-Haired Hound (ISTK) n = 14, Istrian Wire-Haired Hound (ISTO), n = 7, Yugoslavian Shepherd Dog - Sharplanina (SAR) n = 12, Serbian Hound (SRP) n = 8, Bosnian and Herzegovinian/Croatian Shepherd Dog - Tornjak (TOR) n = 6, and Serbian Tricolour Hound (TRO) n = 11. SAR and TOR are large-sized Livestock Guardian Dog breeds classified in Group 2 by The Fédération Cynologique Internationale (FCI), for which no working trial is required. The remaining breeds investigated in this study belong to FCI Group 6 as medium-sized scenthounds, for which a working trial is required.

The age of the dogs ranged from 6 months to 10 years, with 90% of the individuals being young animals and mature adults up to six years of age. Both sexes were represented across all breeds, with a total of 36 females and 49 males. There were no related individuals among the dogs included in the study.

Blood samples were obtained at dog shows attended by dogs from various regions across the country, as well as from neighboring countries. All animals were clinically healthy and in good condition. From each dog, two samples (whole blood with EDTA and serum) were collected via cephalic vein puncture. Immediately after sampling, the samples were transported to the laboratory following the cold chain principle. The whole blood and serum samples were kept in the refrigerator overnight. The next day, the samples were frozen and stored at -20°C until analyses. Analyses were performed within two months from the sampling date. Preparation and storage, as well as DNA isolation and preparation for droplet digital PCR (ddPCR), were carried out in the laboratories of the University of Sarajevo - Veterinary Faculty.

The study protocol was approved by the Ethics Committee of the University of Sarajevo - Veterinary Faculty (approval number 07-03-136-2/25). Verbal informed consent was obtained on-site from the dog owners, witnessed by at least two members of the research team. All routine venipuncture procedures were conducted in the presence of the dog’s owner, without any special pre-procedural measures, such as anesthesia or analgesia.

### Serological tests

The serum samples were thawed immediately before analysis. Using IDEXX’s Catalyst One apparatus (IDEXX Laboratories, Inc., Westbrook, Maine, USA), glucose and amylase concentrations in the serum were determined with IDEXX Catalyst GLU and IDEXX Catalyst AMYL tests according to the manufacturer recommendations. The obtained values of serum amylase concentration (U/L) were converted into units of amylase activity (μkat/L; microcatalytic units per liter) by multiplying by the factor 16.67/1000.

### DNA extraction and *AMY2B* copy number assay

DNA was extracted from whole blood samples using the DNeasy Blood & Tissue Kit (Qiagen, Hilden, Germany) according to the manufacturer’s recommendations for this type of sample. The final elution of DNA was performed with 100 µ L Buffer AE.

The determination of *AMY2B* gene copy number was performed at BIOCEV laboratories in the Czech Republic. ddPCR system was used to count copies of the target gene, along with the C7orf28b reference gene, following the protocol outlined by Axelsson et al. [3]. DNA concentration was assessed prior to ddPCR in order to determine and adjust the sample concentration. Concentration analysis was conducted using a NanoDrop device (ThermoFisher), and samples with concentrations between 5–40 ng/μL were selected for testing. All samples were processed with restriction endonuclease digestion, and the total number of copies of the *AMY2B* gene was determined. In a reaction with a total volume of 10 μL, 5 μL of DNA was treated with 1 U/μL BamHI (NEB) restriction enzyme in CutSmart enzyme buffer. The samples were incubated for 60 minutes at 37°C. In the ddPCR reaction volume of 20 μL, 2 μL of digested matrix was used. Following the manufacturer’s instructions, droplets were produced, with an average of 11,000 (9,000–15,000) per reaction. PCR amplification for testing in dogs was performed using a C1000 device (BioRad) with the following programs: denaturation at 95°C for 10 minutes, 40 cycles of denaturation at 95°C for 30 seconds, hybridization at 60°C for 60 seconds of elongation, and finally stabilization of droplets at 98°C for 10 minutes, followed by cooling at 4°C. The PCR reaction was carried out with 450 nM primer and 250 nM probe [10]. *AMY2B* copy number was determined using Quantasoft ver. 1.6.6.0320.

### Statistical analysis

In the statistical analysis of the results, a one-way ANOVA was applied to determine whether the studied parameters differ among dog breeds, along with a post hoc Tukey test to examine the significance of differences between breeds when the breed showed a significant effect. Pearson’s correlation coefficients were calculated to test for associations between the parameters. A two-tailed t-test was used to examine differences between sexes, as well as differences between the two major phylogenetic groups of dogs in this study (guardians and hounds). P values less than 0.05 were considered statistically significant. All statistical analyses were performed using the software Minitab 17.

## Results

**Fig 1:**
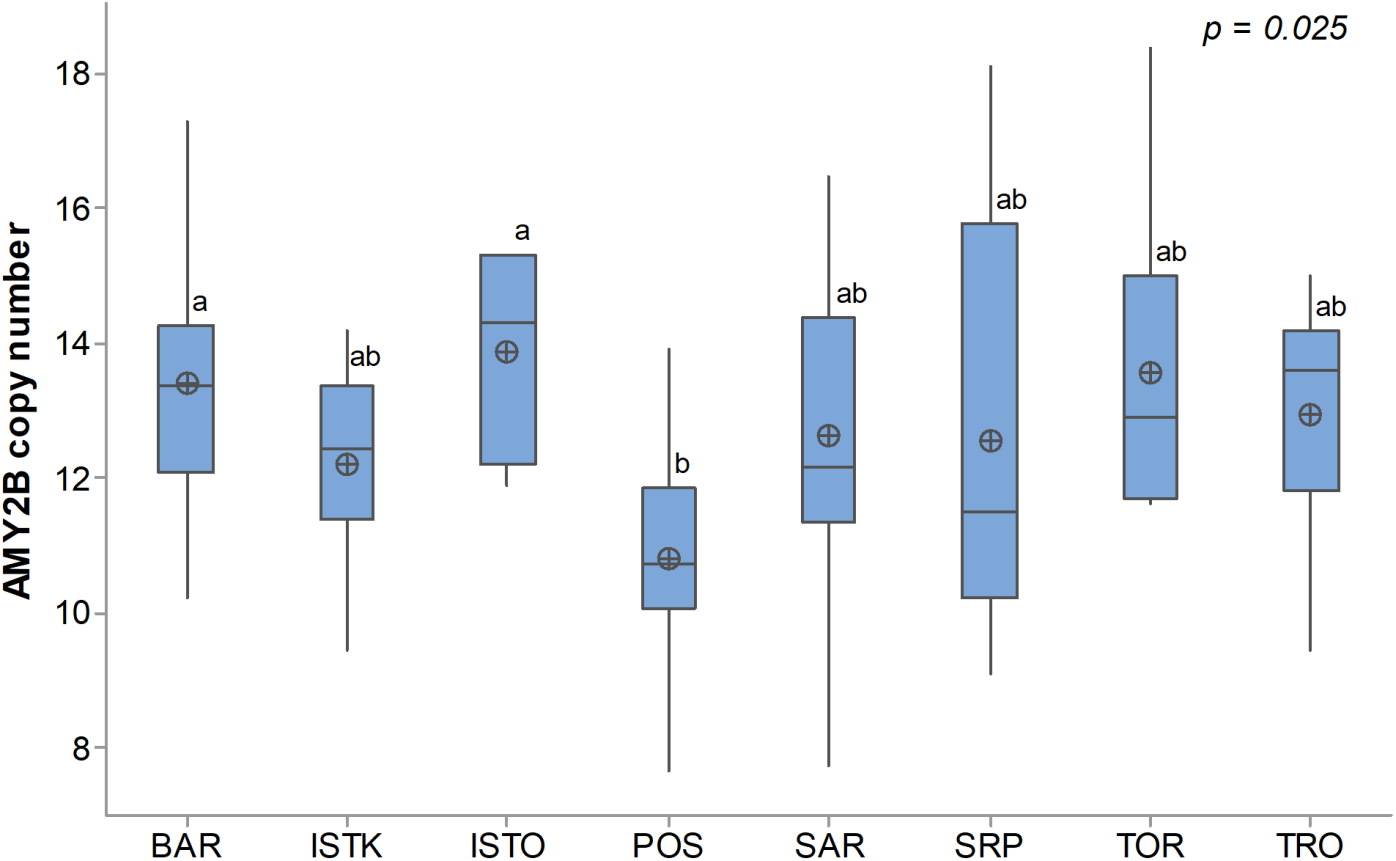
Diploid copy number of *AMY2B* gene in Balkan dog breeds. BAR - Barak, ISTK - Istrian Short-Haired Hound, ISTO - Istrian Wire-Haired Hound, POS - Posavatz Hound, SAR - Sharplanina, SRP - Serbian Hound, TOR - Tornjak, TRO - Serbian Tricolour Hound. ^ab^ - Different superscript letters indicate statistical significance (p < 0.05) of differences between means.

The diploid number of *AMY2B* gene copies in 85 dogs from eight breeds in this study ranged from 7.7 to 18.4. All examined dogs, except for two, carried more than eight copies of the gene. The mean copy number across all dogs was 12.4 ± 2.2. Analysis of variance revealed a significant breed-related difference (p = 0.025). Specifically, these differences were observed between the ISTO and POS breeds, as well as between the BAR and POS breeds (Fig 1). The ISTO breed exhibited the highest mean *AMY2B* copy number (13.9 ± 1.5), while the POS displayed the lowest (10.8 ± 1.5). The greatest variation in copy numbers was observed among SRP dogs, ranging from 9.1 to 18.1, whereas ISTO dogs exhibited the least variability, ranging from 11.9 to 15.3. The difference between females (12.4 ± 1.6) and males (12.8 ± 2.5) was not significant (p = 0.377), nor was the difference between guardians (12.9 ± 2.4) and hounds (12.5 ± 2.1) (p = 0.466).

**Fig 2:**
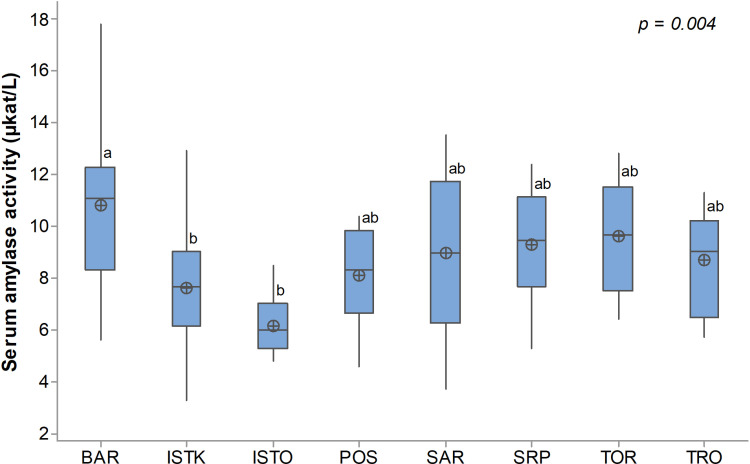
Serum amylase activity in Balkan dog breeds. BAR - Barak, ISTK - Istrian Short-Haired Hound, ISTO - Istrian Wire-Haired Hound, POS - Posavatz Hound, SAR - Sharplanina, SRP - Serbian Hound, TOR - Tornjak, TRO - Serbian Tricolour Hound. ^ab^ - Different superscript letters indicate statistical significance (p < 0.05) of differences between means.

Serum amylase activity ranged from 3.3 to 17.8 µkat/L, with a mean value of 8.7 ± 2.6. A significant interbreed difference was noted (p = 0.004). The BAR breed exhibited the highest mean value (10.8 ± 3.0), which was significantly higher than those observed in the ISTK (7.6 ± 2.4) and ISTO (6.2 ± 1.3) (Fig 2). The remaining five breeds showed comparable means both among themselves and when compared to the three breeds mentioned earlier.

**Fig 3:**
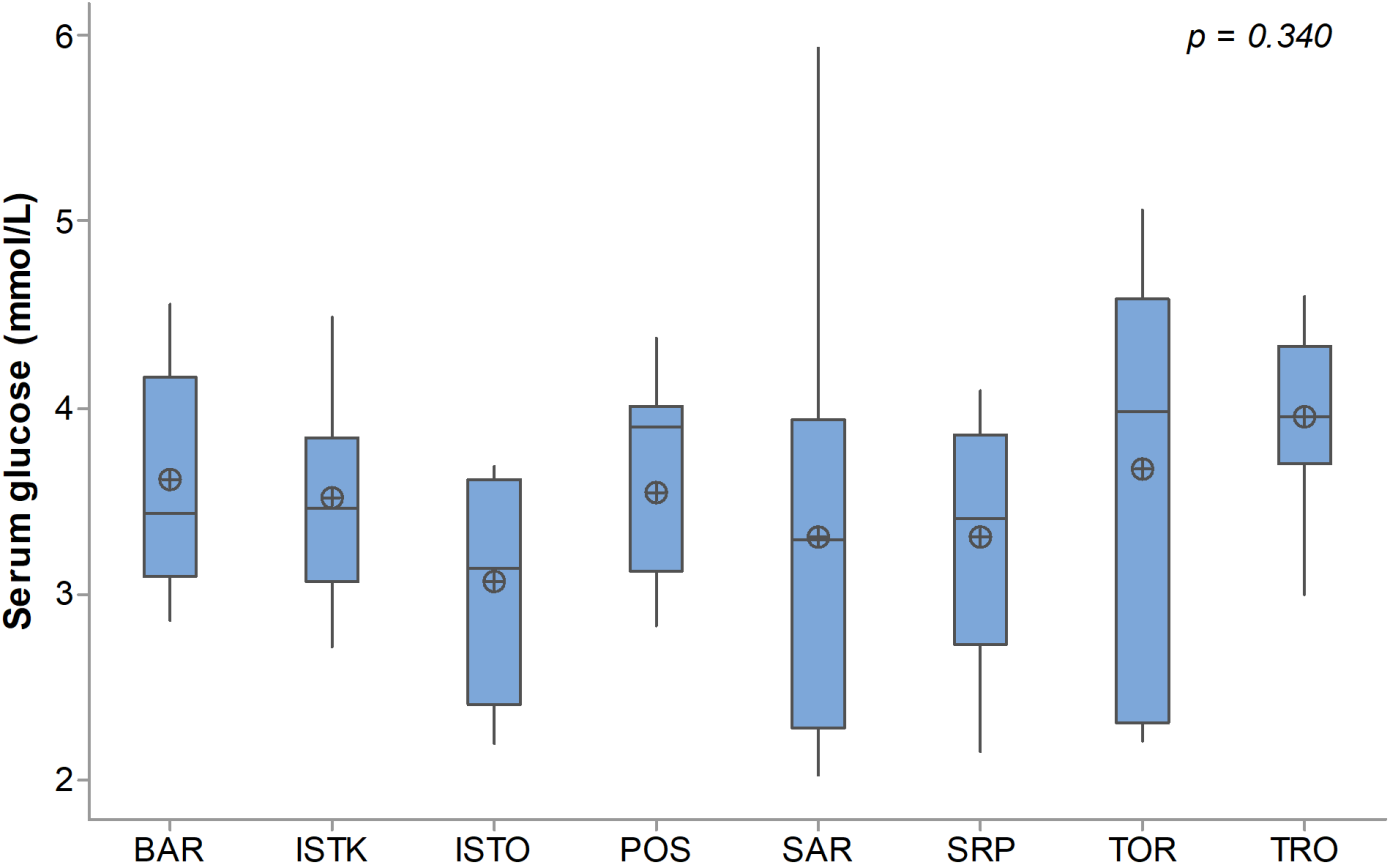
Serum glucose content in Balkan dog breeds. BAR - Barak, ISTK - Istrian Short-Haired Hound, ISTO - Istrian Wire-Haired Hound, POS - Posavatz Hound, SAR - Sharplanina, SRP - Serbian Hound, TOR - Tornjak, TRO - Serbian Tricolour Hound.

While the levels of serum glucose content varied widely and showed numerical differences among the breeds (Fig 3), statistical significance for these variations could not be confirmed (p = 0.340). The TRO breed exhibited the highest mean glucose value (3.95 ± 0.50 mmol/L), whereas the lowest was recorded in the ISTO (3.06 ± 0.57 mmol/L). Individual glucose values among all dogs ranged from 1.08 to 5.94 mmol/L, with an overall mean of 3.51 ± 0.77 mmol/L.

The correlation coefficients suggest no significant association between the number of *AMY2B* gene copies and serum amylase activity, as well as between *AMY2B* copy numbers and glucose content (Table 1).

**Table 1:** Correlation coefficients between amylase activity, serum glucose content and *AMY2B* gene copy number in Balkan dog breeds (n = 85).

	Glucose	*AMY2B*
**Amylase**	r = 0,099 *p* = 0,365	r = 0,153 *p* = 0,161
**Glucose**		r = -0,098 *p* = 0,372

r - Pearson correlation coefficient; *p* - statistical significance of the correlation coefficient.

## Discussion

The number of copies of the *AMY2B* gene found in dogs in our study generally corresponds to the position occupied by European dogs within the global distribution of *AMY2B* copy numbers, as demonstrated by Arendt et al. [6]. The authors showed across 46 dog breeds and 114 native dogs that the average copy number of this gene is higher in prehistoric agrarian regions of the world (ranging from 8.5 to 11.8 copies) compared to dog lineages originating from non-agrarian regions such as Arctic America (3.7) and Arctic Asia (6.4), notably Australia (2.2). Among agrarian geographic regions, European dogs exhibit the highest number of *AMY2B*, with an average of 11.8 copies [6], a value very close to the mean of 12.4 copies found in Balkan dogs in our study.

Agrarian practices in the Balkans can be traced back to the Neolithic age [9], which is also considered the time of expansion of the *AMY2B* gene in dogs [8]. Ollivier and colleagues found that even in some ancient dog breeds originating from prehistoric agrarian areas, there was a significant expansion of the *AMY2B* gene (more than eight copies) during this period [8]. In our study, 83 out of 85 dogs exhibited more than eight copies of the gene, indicating that the examined breeds have historically experienced strong selection pressure for adaptation to starch-based foods. The entire Balkan region is part of an area with early developed agriculture [8,9], primarily the culture of growing cereals rich in starch, especially wheat and barley, as well as other crops such as lentils [9]. These crops likely served as an additional food source for dogs, which could explain the expansion of the *AMY2B* gene observed in the dogs examined in our study. Research has shown that the expansion of *AMY2B* copy numbers correlates with the spread of prehistoric agriculture [6]. Additionally, according to research [8], this expansion reflects a local adaptation that enabled dogs to thrive on a starch-rich diet, suggesting a biocultural coevolution of dog genes and human culture. Intensive cultivation of cereals and other agricultural crops in the Balkans, including the territory of Bosnia and Herzegovina, was also recorded in the Roman era [11]. The conquered indigenous Illyrian people in that region had a rich hunting tradition, which declined during the Roman period. However, agriculture experienced significant expansion, particularly with the increased cultivation of bread grains, including rye and wheat, as well as winter grains [12]. These changes in agricultural and hunting practices may have further influenced the expansion of the *AMY2B* gene in dogs.

The *AMY2B* copy numbers in Balkan dogs in this study were similar to those found in most modern breeds [4], but slightly higher than the other results [13]. Certain Chinese dog breeds traditionally consuming high-starch diets typically had 7–14 copies of this gene, with the majority (74%) having at least 10 copies [13]. In our study, 92% of dogs had at least 10 copies of *AMY2B*. Additionally, breeds such as Barak, Tornjak, and Istrian Wire-Haired Hound consistently exhibited diploid copy numbers above 10, as previously observed in German Shepherds, English Springer Spaniels [4], Poodles, Border Collies, Tazys, and others [6].

We found that *AMY2B* copy numbers vary considerably in dogs, both within and across breeds, as demonstrated in earlier studies [3–5,8]. Such variability in gene copy numbers in our dogs can be considered an expected finding due to the number of breeds included in the study. *AMY2B* copy number varied across all breeds, with notable variation observed in Serbian Hounds (12.6 ± 3.2), contrasting with the relatively homogeneous copy numbers found in Istrian Wire-Haired Hounds (13.9 ± 1.5). Arendt et al. similarly observed high variability at this locus specifically in Beagles, whereas Polish Lowland Sheepdogs were relatively homogenous [4]. Reiter et al. [13] reported that breeds traditionally consuming low-starch diets (Siberian Husky, Alaskan Malamute, Japanese Dogs) exhibited significantly higher variation (40–80%) and lower average copy number compared to breeds consuming high-starch diets (Shar Pei, Pekingese; variation 16–24%). However, such an inverse relationship between *AMY2B* copy number and variability was not observed in our study. Specifically, variability was lowest within the breed with the highest number of copies (Istrian Wire-Haired Hound), but the highest variability was not found in the breed with the lowest number of copies (Posavatz Hound; 10.8 ± 1.5), but instead in the Serbian Hound.

In the study of Arendt et al. [4] breed origin explained more than 50 percent of copy number variability in *AMY2B*, the remaining depends on individuals. The breed in our study also had a significant impact on the number of gene copies (p = 0.025). The highest means of the *AMY2B* were observed in Istrian Wire-Haired Hound and Barak, which were significantly higher compared to Posavatz Hound, where the lowest number of copies was found. The remaining five breeds did not show significant differences either among themselves or compared to the three breeds mentioned above. This finding confirms that copy number variation can differ both between breeds and among individuals within the same dog population, even within a narrow geographical area [3,5,8]. Since there is no reliable data available on the historical age of the breeds included in our study or when they transitioned to high-starch diets, it is not possible to logically explain the origin of these differences.

As hypothesized by others, pancreatic amylase activity is associated with *AMY2B* copy numbers within the dog population. Consequently, the ability to digest starch may vary among dogs due to variation in *AMY2B* across breeds and within individuals. This suggests that certain dogs, and potentially specific breeds, may exhibit greater efficiency in starch digestion compared to others [3,4]. Such variations could have practical implications for diet composition, food utilization, and overall animal health. Serum amylase activity in dogs in our study ranged from 3.3 to 17.8 μkat/L, which is a wider range than that reported by Axelsson et al. [3]. Since most of the sampled dogs were young animals and mature adults brought to dog shows, we assume that none of them suffered from pancreatitis, which would lead to a pathological increase in serum amylase. This assumption is further supported by interviews conducted with the owners, who denied any history of the disease. However, in 36 samples, the amylase levels were below the lower reference range (8.3–25.0 μkat/L).

No correlation was found between *AMY2B* copy numbers and serum amylase activity in our study (r = 0.153; p = 0.161). Furthermore, the means of amylase activity across dog breeds did not follow the same breed-dependent pattern observed for *AMY2B* copy numbers. The highest mean amylase activity was found in the Barak, which was significantly higher compared to both Istrian dog breeds in which the lowest amylase activity was observed. It is possible that Istrian dogs traditionally had different eating habits because they originate from the coastal region of Croatia, where they likely consumed fish or fish remains more frequently. Interestingly, the Istrian Wire-Haired Hounds had the highest number of copies of the *AMY2B* gene among the tested Balkan breeds. Therefore, the finding of the lowest amylase activity in this breed is unexpected and inconsistent with earlier research [4]. Arendt and colleagues reported that amylase activity in dogs increases linearly with *AMY2B* copy number, a finding that was not confirmed in our study. Concerning specific gene impact, the same authors also found that serum amylase activity increases by 5.4% with each additional copy of the *AMY2B* gene. This warrants the need for further investigation.

Although serum amylase activity correlated with the number of *AMY2B* copies in dogs in a previous study, only a small proportion of its variance was explained by the variation in copy numbers (R^2^ = 14.8%) [4]. The authors suggested that additional factors play a significant role in explaining the majority of amylase variation, potentially including other conditions apart from pancreatitis, as well as dietary habits, age, and circadian rhythm. Furthermore, serum amylase activity is one of the most variable biochemical analytes in dogs, depending also on factors such as breed, sex, and neutering status [14]. Therefore, Arendt et al. [4] advise caution when interpreting these activity measures as a direct indicator of inherited starch-handling ability. They also suggest that future studies using larger cohorts under controlled conditions may enable the establishment of copy-number-specific reference values for serum amylase activity, facilitating the detection of abnormal activity.

A potential link between *AMY2B* copy number and susceptibility to developing diabetes mellitus in dogs was investigated, but no association was found [4]. However, in a porcine model, it has been demonstrated that pancreatic amylase actively participates in glucose absorption/utilization and in the maintenance of postprandial glucose homeostasis [15]. In our study, no statistical correlation was found between serum glucose levels and amylase activity, nor between glucose levels and the number of *AMY2B* copies. Furthermore, a significant number of dogs exhibited hypoglycemia, which could have further complicated the detection of potential correlations. The dogs were brought from different cities and neighboring countries to dog shows, and the transport process itself, along with its conditions, may induce stress in the animals. They were also not fed in the morning before and while being shown. During states of stress and fear, additional activities consume glucose and may contribute to hypoglycemia [16]. These changes likely contributed to the inability to detect a correlation between amylase activity and serum glucose (r = 0.099; p = 0.365).

## Conclusion

Our research suggests that the early development of agriculture in the Balkans likely contributed to an increase in the number of *AMY2B* genes, representing an evolutionary advantage in dogs through positive selection. Due to this advantage, dogs originating from the Balkans, similar to most European breeds, could more easily withstand the evolutionary pressure resulting from the starch-rich diet to which they had to adapt. Significant differences in *AMY2B* gene copies and serum amylase activity were observed among dog breeds in this study. However, the expected correlation between these variables was not confirmed, which was inconsistent with earlier studies. This further supports the recommendation that additional studies under controlled conditions are needed to establish and clarify the relationships between the *AMY2B* copy number, pancreatic amylase activity, and glucose metabolism in dogs.
